# The role country of birth plays in receiving disability pensions in relation to patterns of health care utilisation and socioeconomic differences: a multilevel analysis of Malmo, Sweden

**DOI:** 10.1186/1471-2458-6-71

**Published:** 2006-03-16

**Authors:** Anders Beckman, Anders Hakansson, Lennart Rastam, Thor Lithman, Juan Merlo

**Affiliations:** 1 Department of Clinical Sciences, Lund University, University Hospital, S-205 02 Malmo, Sweden; 2 Regional Office, Scania County Council, Lund, Sweden

## Abstract

**Background:**

People of low socioeconomic status have worse health and a higher probability of being granted a disability pension than people of high socioeconomic status. It is also known that public and private general physicians and public and private specialists have varying practices for issuing sick leave certificates (which, if longstanding, may become the basis of disability pensions). However, few studies have investigated the influence of a patient's country of birth in this context.

**Methods:**

We used multilevel logistic regression analysis with individuals (first level) nested within countries of birth (second level). We analysed the entire population between the ages of 40 and 64 years (n = 80 212) in the city of Malmo, Sweden, in 2003, and identified 73% of that population who had visited a physician at least once during that year. We studied the associations between individuals and country of birth socioeconomic characteristics, as well as individual utilisation of different kinds of physicians in relation to having been granted a disability pension.

**Results:**

Living alone (OR_women _= 1.72, 95% CI: 1.62–1.82; OR_men _= 2.64, 95% CI: 2.46–2.83) and having limited educational achievement (OR_women _= 2.14, 95% CI: 2.00–2.29; OR_men _= 2.12, 95% CI: 1.98–2.28) were positively associated with having a disability pension. Utilisation of public specialists was associated with a higher probability (OR_women _= 2.11, 95% CI: 1.98–2.25; OR_men _= 2.16, 95% CI: 2.01–2.32) and utilisation of private GPs with a lower probability (OR_men _= 0.76, 95% CI: 0.69–0.83) of having a disability pension. However, these associations differed by countries of birth. Over and above individual socioeconomic status, men from middle income countries had a higher probability of having a disability pension (OR_men _= 1.61, 95% CI: 1.06–2.44).

**Conclusion:**

The country of one's birth appears to play a significant role in understanding how individual socioeconomic differences bear on the likelihood of receiving a disability pension and on associated patterns of health care utilisation.

## Background

Sweden has a general welfare policy that guarantees financial security and social rights to all citizens [1]. Included in this system is the provision of a disability pension for those between the ages of 30 and 64 who, for medical reasons, are incapable of working or supporting themselves financially [2].

A number of studies have investigated the association between different measures of health status [3,4], medical conditions [5], and receiving disability pensions. Studies of health care utilisation after being granted a disability pension have also been made, with varying results [5-7]. It is known that people of low socioeconomic status have worse health and higher health care requirements than people of high socioeconomic status [8-11]. Therefore, they have a higher probability of eventually being in need of a disability pension [3]. The area where one resides [12] and its socioeconomic characteristics [13] also seem to affect an individual's likelihood of receiving a disability pension.

Even if disability pensions were granted solely on objective medical grounds, the probability of obtaining a disability pension may depend on other types of factors, operating on different levels [12,14]. It is known that a patient's ability to communicate information and articulate needs influence the provision of health care [15] and disability pensions [16]. Based on an analysis of sick leave certifications, it has also been observed that different types of physicians assign different periods of sick leave for the same diseases [17,18]. Few studies, however, have investigated the influence of ethnicity in this context [19-22].

Members of a given ethnic group tend to identify themselves and be identified by others on the basis of specific characteristics differentiating them from other groups. People who are born in the same country and who share a number of economic, social, and cultural characteristics in addition to a common geographic origin and language, may exhibit a related probability of receiving disability pensions. In a life-course approach, it is possible that global social circumstances related to the economy of the country of one's birth act to condition individual experiences while growing up. These experiences may, in turn, affect individual health, behaviour, and attitudes, and may find expression years later, after immigration to a new country. The original country of birth may, therefore, bring about patterns of health care utilisation [23], including the likelihood of receiving a disability pension, that are common to those individuals born there.

Sweden offers an ideal scenario for investigating cultural determinants of health care utilisation. Equitable access to health care is ensured by law [24]. Ninety-four percent of the health care system originates from state and county finances that support providers in both the public and private sectors. This universal health insurance seeks to allocate resources on the basis of need, rather than such factors as gender, socioeconomic position, or country of birth [25]. This societal funding of both public and private health care sectors removes fees for services as a major contributing factor by making costs similar between sectors. Also, a direct access to health care, with no gatekeeper function exercised by general practitioners, makes individual choice of provider possible. Patterns of utilisation of specific health care services that may be influenced by country related factors (e.g., learned patterns and expectations) or by an immigrant's interaction with Swedish society can then be studied without being confounded by economical or administrative barriers.

In the presence of this intra-country correlation, multilevel regression analysis (MLRA), for both statistical and epidemiological reasons, appears to be an appropriate methodological approach for investigating the influence country of birth may have exercised on those currently holding disability pensions. MLRA allows us to quantify the role that country of birth plays for understanding individual differences among disability pension recipients [26,27] and also correctly assess the association between country of birth characteristics (e.g., the economy of that country) and the probability that someone born there will one day receive a disability pension in Sweden [28].

Studying the total population of the city of Malmo, Sweden, we sought to assess the role of the country of one's birth in relation to the probability of having a disability pension. Our intention was to investigate possible associations between individual socioeconomic characteristics (i.e., living alone and level of educational achievement) and being granted a disability pension. In addition, by focusing on those individuals who had had some contact with the Swedish health care system, we wished to determine if an association existed between the kind of physician consulted (i.e., public and private general practitioners, and public and private specialists) and being granted a disability pension.

## Methods

### Study population

Our study population consisted of all 80 212 individuals aged 40 to 64 years (from 146 different countries) who resided in Malmo, Sweden, during 2003. (Malmo is the largest city in the County of Scania, in southern Sweden, with 257 455 inhabitants as of January 1, 1999.) Of the 80212 people studied, 33% were foreign-born men (n = 40 471) and 31% foreign-born women (n = 39 748). We also identified 73% of the population (58 848/80 212) who had visited a physician at least once in 2003. Of this group, 32% were foreign-born men (n = 26 599) and 31% foreign-born women (n = 32 249).

The present analysis is based on the 2003 County of Scania Register for Resource Allocation, which includes, among other variables, age, gender, marital status, education, income, disability pension, and country of birth, as well as detailed information on health care utilisation for each individual in the county. The Regional Ethics Review Board in Lund, and the Data Safety Committee at Statistics Sweden, approved the use of this database. The information was handled in such a way as to preserve the anonymity of the subjects. Analyses were performed at the individual level, but results always presented in the aggregate.

### Outcome variable

A disability pension may be granted for medical reasons to a person who has lost 25% to 100% of their working capacity. For our purposes, the beneficiary of a disability pension is any individual who has been granted such a pension, irrespective of their degree of disability.

### Individual characteristics

We considered those people who were single, separated, or widowed as "living alone". This definition did not take into account unmarried couples or people sharing the same household – a factor that might produce misclassification and lead to underestimating the association between living alone and having a disability pension. Formal schooling of nine years or less was categorised as "low educational achievement". Those with further schooling were labelled as having "high educational achievement". Age was considered a contiguous variable and centred on the median. Since the association between age and having a disability pension may not be linear, age-squared was also included in the models. Utilisation of health care (in the form of visits to doctors, irrespective of the type of provider, but excluding hospitalisations) was classified as either "yes" or "no". Those who visited a physician at least once were categorized according to the type of provider they consulted: public general practitioner, public specialist, private general practitioner, or private specialist. An individual who visited different types of physicians during 2003 received multiple classifications.

### Country of birth (i.e., contextual) variables

The socioeconomic characteristics of one's country of origin, over and above those of the individual, may have conditioned the likelihood of receiving a disability pension. We tested this hypothesis by using the World Bank Classification of Country Economies as a contextual variable [29] in which countries are classified according their gross national income (GNI) per capita, using the World Bank Atlas method. The GNI categories employed are low, lower middle, upper middle, and high income. We merged the first two into a single category designated "low income country", and used the high income country category as a reference in the comparisons.

### Statistical and epidemiological methods

In order to account for a possible modification of effects due to gender, we stratified the study population into males and females.

Due to the hierarchical structure of the data, with individuals nested within countries of birth, and the possibility of intra-country correlation regarding the likelihood of having a disability pension, we applied multilevel logistic regression [26,30,31]. Of the 146 different countries of birth that made up our study population, 66 countries were represented by fewer than ten individuals. However, this disproportion can be satisfactorily handled by multilevel regression analysis [31].

We investigated disability pensions for the entire population and separately for those who had contact with physicians. We then established four consecutive multilevel models. In the first or empty model (A), the probability of having a disability pension was only a function of an individual's country of birth and was modelled by a random intercept. Our second model (B) included the individual variables of age, education, and marital status. The third model (C) considered an individual variable for utilisation of health care. Finally, the fourth model (D) expanded the third model to include the contextual variable "World Bank Classification of Country Economies". Models B and C also allowed us to study the interaction between country of birth and individual variables (educational achievement, marital status, and the variable for utilisation of health care) by allowing the regression coefficients of the individual level variables to be random at the level of country of birth. Such models indicate whether the bearing of individual variables on disability pensions differ by country of birth (i.e., for individuals from some countries, living alone or consulting a public general practitioner may be associated with a higher probability of having a disability pension, whereas in other countries the reverse may be true).

By this general strategy we were able to quantify differences between various countries of birth (model A) and estimate the role played by individual characteristics of people from each nation, as well as quantify possible cross-level interactions between marital status, education, and country of birth (model B). The third model (C) assessed the relationship between consulting different providers and having a disability pension, and also indicated possible cross-level interactions between individual patterns of visiting different types of physicians and one's country of birth. Finally, the last model (D) indicated whether the economic circumstances in one's country of birth, over and above individual conditions, were associated with being granted a disability pension in Sweden.

We appraised the association of the variables studied and having a disability pension by odds ratios (OR) with a 95% confidence interval (CI), as obtained from the regression coefficients (standard error).

In order to quantify the influence of country of birth for having a disability pension, we computed the median odds ratio (MOR) [32]. With this method, the variance at the second level (country of birth) is translated into the well-known OR scale. The MOR could represent how much (in median) the likelihood of having a disability pension would increase if an individual had been born in a country whose inhabitants had a greater probability of receiving disability pensions. If the MOR is equal to one, country of birth does not condition an individual's likelihood of having such a pension.

In the presence of slope variance for marital status or level of education (model B), or the variable for utilisation of health care (model C), the country of birth variance becomes a function of individual marital status, education, or physician utilisation (e.g., disability pension differences between countries of birth may be much greater for married individuals than for those living alone, or lower for those who visit a private general practitioner than for those who visit other physicians). Therefore, we calculated the variance between countries of birth as a function of individual variables [33-35], and expressed this variance on the OR scale by means of the MOR [36].

MLwiN software, version 2.0 [34], was used for the analyses. Parameters were estimated using the Markov Chain Monte Carlo (MCMC) procedure. The Deviance Information Criterion (DIC) was used as a measure of how well our different models fit the data. A lower value on the DIC indicated a better fit of the model [37].

## Results

Table 1 indicates that 11% of the men and 16% of the women between the ages of 40 and 64 who lived in Malmo in 2003 were granted disability pensions. However, this figure was somewhat higher (14% for men and 18% for women) among those who had had at least one contact with a health care provider in 2003. Individuals from middle income countries had the highest likelihood of having disability pensions. Low educational achievement was less common among individuals from countries with a flourishing economy, but the percentage of people living alone increased as a country's economy improved. A greater number of women than men (81% vs. 66%) had seen a physician one or more times in 2003. Public GPs were more frequently consulted by people from countries with poorer economies. Women from wealthier countries preferred private specialists to a greater extent than men from the same countries.

Compared to the entire population (Table 1), individuals who had disability pensions (Table 2) more frequently lived alone, had less education, and were in greater contact with health care providers. In general, such individuals tended to use public physicians.

### Measures of association (fixed effects)

As seen in Table 3, model B, living alone and having low educational achievement were clearly associated with having disability pensions for both men and women. Compared with married and individuals with high educational achievement, those living alone and having low educational achievement had a higher probability of being granted a disability pension. However, marital status seemed a more relevant factor for men and educational level for women.

In Table 3, model C, we also note that the OR of receiving disability pensions was high among those who had paid at least one visit to a physician in 2003, both in the case of women (OR 2.45, 95% CI: 2.21–2.71) and men (OR 2.09, 95% CI: 1.93–2.27).

We observed (Table 3, model D) that over and above individual age, educational level, or marital status, men from middle income countries had a higher probability of having a disability pension, and this association was lower and not conclusive for women (OR_men _= 1.61, 95% CI: 1.06–2.44; OR_women _= 1.34, 95% CI: 0.82–2.19).

Table 4 represents a similar analysis as Table 3, but this population is restricted to those who have had at least one contact with a physician in 2003. The pattern of association between marital status, education, and country economy in relation to holding a disability pension was similar to that observed in the analysis of the whole population. The association between utilisation of different physicians and having a disability pension was rather heterogeneous. In the case of both genders, using public specialists was clearly associated with a higher probability of having a disability pension (OR approximately 2). We found a slightly higher prevalence of disability pension recipients among women who had visited public GPs, but not in men (OR_women _= 1.23, 95% CI: 1.15–1.32; OR_men _= 1.09, 95% CI: 1.01–1.18). However, having a disability pension seemed to be less frequent among individuals visiting private GPs than among those consulting other physicians, especially for men (OR = 0.76, 95% CI: 0.70–0.83). Disability pensions were not associated with the utilisation of private specialists in either women or men (OR_women _= 1.04, 95% CI: 0.96–1.09; OR_men _= 1.04, 95% CI: 0.96–1.12).

Over and above the individual characteristics considered in the analysis, being born in a middle income country (Table 4, model D) increased the probability that a man would receive a disability pension (OR = 1.58, 95% CI: 1.08–2.33).

### Measures of variation (random effects)

Tables 5 and 6 show the country of birth variance obtained in the models presented in Tables 3 and 4. Overall, country of birth variance in disability pensions (Table 5, model A) was higher for women than for men (MOR = 2.17 and 1.84, respectively), and this pattern persisted when the analyses were restricted to individuals who sought health care (Table 6, model A).

In model B, Table 5, consideration of the individual composition of countries with regard to marital status and educational achievement improved the fit of the model (as seen by the reduction in the DIC value).

In model C, Table 5, allowing for slope variance for the variables of marital status and educational level also improved the fit. Furthermore, because of the existence of slope variance, country of birth differences in disability pensions became a function of individual marital status; the same was true for the education variable. Country of birth variance appeared to be greater (i.e., a higher MOR) among married individuals than among those living alone, especially for women (MOR_women _2.08 vs. 1.64; MOR_men _1.81 vs. 1.50). We also observed greater discrepancies by countries of birth (i.e., a higher MOR) among those with less schooling than for individuals with more education.

When country of birth variance in disability pensions became a function of visiting a physician, we observed a higher MOR for those who had not sought health care than for individuals who had consulted a physician at least once, a fact that was more conspicuous in men than women.

Table 6, model C, indicates that when country of birth variance in disability pensions became a function of utilisation of different providers, the country of birth differences for those who had visited a public specialist (MOR_women _1.76) were lower than for those who had not (MOR 2.10). An opposite pattern was seen for individuals who had utilised private specialists, i.e., their MOR was higher than those who had not.

## Discussion

We sought to quantify the role of country of one's birth in understanding the individual probability of being granted a disability pension in the current country of residence (i.e., Sweden). We also examined the association between individual marital status, educational level, and utilisation of health care on the one hand, and disability pensions on the other, weighing whether this association was modified by country of birth. Moreover, we considered whether the economic circumstances of one's country of birth influences the likelihood of receiving a disability pension in the present country of residence (i.e., Sweden), independent of individual socioeconomic characteristics. The simultaneous analysis of these essential research questions represented a methodological challenge that was resolved by applying multilevel regression analysis.

Analogous to what we previously found regarding socioeconomic factors and the utilisation of specific health care providers [23], we observed a clear association between having less education and living alone, on the one hand, and receiving a disability pension, on the other. These associations were expected since it is know that people with low socioeconomic status who live alone have worse health than their high socioeconomic status counterparts and, therefore, have a higher likelihood of being granted a disability pension. However, it was striking to find that these associations were modified by the country of one's birth.

Using the information provided by the MOR as a measure of country of birth variance, we found that country of birth played a greater role than other individual variables when it came to understanding individual differences in the probability of having a disability pension. For example, the individual odds ratio for women living alone to receive a disability pension was 1.62 (Table 4, model B). However, the influence of country of birth was a MOR of 1.90 (Table 5, model B). This contextual effect was more pronounced for women than for men (Table 5). These gender specific effects may express different gender roles and expectations in different countries. An aspect that need more (qualitative) investigation in order to tailor effective public health intervention directed to reduce inappropriate granting of disability pensions [38].

Random slope analysis significantly contributed to understanding the part played by country of birth in the association between socioeconomic position and disability pensions. From a statistical point of view, the inclusion of a random slope parameter increased the fit of the models, as indicated by a lower DIC value. From an epidemiological perspective, the existence of slope variances indicates that socioeconomic gradients were steeper for some countries of birth than for others. Because of this phenomenon, country of birth played a different role for understanding individual differences in the probability of having disability pensions among married people and those with minimal education. It is possible that individuals with greater education and those living alone share values and circumstances that are independent of their country of birth, which would explain the lower MOR for these groups. One might speculate that cultural aspects in some countries dissuade married people from seeking disability pensions, while in other cultures the opposite may be true. We need further qualitative studies in order to comprehend these reasons. In any case, conventional measures of association between socioeconomic factors and disability pensions may be misleading if contextual elements related to country of birth are not considered. This consideration would be of substantial importance in the analysis of health care [39].

We also found that, especially for men, the economic characteristics of one's country of birth influenced the probability of having a disability pension in the new country of residence, i.e., individuals from middle income countries had a greater chance of being granted a disability pension than individuals from more affluent countries (Table 3). However, this contextual effect proved to be not very relevant since the residual country of birth variance was high [36] and the fit of the model did not improve when including this variable.

It is known that having a disability pension is associated with health care utilisation [5,6] – a fact confirmed by our study. Unexpectedly, however, we noted that the OR for having a disability pension was twice as great for those who had consulted public specialists (generally through hospital out-patients clinics) than for those who had utilised other types of physicians. The opposite, i.e., a lower OR for having disability pensions, was true of those who had visited private GPs. The apparent association between utilisation of public specialists and disability pensions might be attributable to patients with more severe diseases seeking out public specialists, since these physicians have closer affiliations with hospitals. Alternatively, it may be that the Social Insurance Agency that grants disability pensions insists on medical certificates issued by public specialists. However, as our study was cross-sectional, we do not know whether utilisation of public specialists starts before or after disability pensions are granted.

Being outside the workforce might have a direct negative effect on an individual's health [40] and result in higher utilisation of specialised care. The negative association between utilisation of private GPs and disability pensions may have an analogous explanation in this context, i.e., the demand for hospital resources and/or stipulations by the Social Insurance Agency. However, if the above were true, the same phenomenon would also be seen among private specialists and public GPs, which was not the case. Rather, the answer might partly lie in differences between public and private providers regarding incentives for certain actions. Some authors have found significant differences in the length of sick leave certificates and the extent of cooperation with the Social Insurance Agency by private or public providers [17]. Although our study cannot answer these questions, it may point out a direction for future investigation.

### Limitations of the study

The present study is based on data that has been gathered for administrative purposes and, therefore, its validity might be questioned. However, Sweden has a long tradition of maintaining population and health care registers, and well-developed systems are in place for recording, storing, and managing this information. The database we studied is used for resource allocation analysis, has been checked for registration errors, and has consistently been handled with professional expertise by the Scania County Council and Statistics Sweden. In this context, country of birth, education, disability pension, and marital status are not self-reported, but are based on official statistics maintained by Swedish authorities, thus increasing the validity of this data. Information on visits to different kinds of physicians is recorded by the county patient administrative system that covers all health care facilities in the Scania region.

We did not use personal income as a proxy for socioeconomic position, since our study was cross-sectional and disability pensions reduce income levels. Moreover, the information available to us on income reflects personal rather than household income, limiting the validity of income as a measure of socioeconomic status: people with low personal incomes may reside in households whose total income is high. This phenomenon is more pronounced for women, whom we were especially interested in studying since they receive a higher percentage of disability pensions than men [41].

Education as a proxy for socioeconomic position has the advantage of being independent of disability pensions, since an individual's level of education is achieved early in life, making the risk of reverse causation less likely. Our database was assembled in 2002 and 2003. Since the year 2000 the education register of Statistics Sweden has implemented quality enhancements that reduce the underestimation of advanced educational levels, while increasing the amount of data available on educational achievement among immigrants.

Because the present database was cross-sectional and we lacked information on how long individuals had been living in Sweden, it is possible that after many years immigrants have a history of disability pension awards and health care utilisation similar to Swedish-born individuals. Therefore, some of the differences between countries of birth could partly reflect disparities in the length of the time immigrants have resided in Sweden.

We had no information on reasons for immigration to Sweden among those we studied, and it is possible that differing reasons might affect the probability of being granted a disability pension. Previous studies have found more sick days and a higher probability of receiving a disability pension recorded for immigrants from countries that traditionally have sent migrant labourers to work in Sweden than from refugee countries [19,20], which could reflect differences in work strain.

## Conclusion

The present investigation has found that the country of one's birth plays a relevant role in understanding individual socioeconomic differences related to being granted a disability pension. As expected, we found that men and women with low educational achievement and those who live alone had a higher probability of having a disability pension, but these associations were modified by one's country of birth. We also found qualitative differences in the relationship of health care utilisation to disability pensions, i.e., a higher probability of having a disability pension among individuals who consulted public specialists and a lower probability for those who visited private GPs.

We believe our investigation presents an innovative methodology for studying socioeconomic and ethnic differences in the distribution of disability pensions. By using a multilevel analytical approach that considered country of birth as a higher level in which people are culturally nested, we applied a statistical and conceptual design that we find more appropriate than classical single-level analysis. Classical analyses usually operate with simple ethnic or geographic categorisations that may be unsuitable for studying the impact of socioeconomic or cultural influences on disability pensions [42]. Conversely, employing a way of classifying individuals within a city according to their country of birth (by incorporating a contextual socioeconomic taxonomy that relies on World Bank criteria for a country's economy) provides a simple approach to analysing factors that may contribute to inequalities in health care determinations among a diverse urban population.

## Competing interests

The author(s) declare that they have no competing interests.

## Authors' contributions

AB performed the statistical analysis and AB and JM conceived of the study, participated in the design, analysis, and drafted the manuscript. AH, LR and TL have revised the manuscript critically for intellectual content. All authors have given final approval of the submitted version

## Pre-publication history

The pre-publication history for this paper can be accessed here:



## Figures and Tables

**Table 1:** Country of birth economic characteristics of individuals 40 to 64 living in Malmo, Sweden 2003 Country economies according to World Bank classification. Values in parentheses are percentages.

	Entire population		Country economy					
			High income		Middle income		Low income	

Women	Total	Sought health care	Total	Sought health care	Total	Sought health care	Total	Sought health care

Number of countries	141	135	32	32	27	25	82	77
Number of individuals	39 747	32 249	29 795	24 272	5 643	4 588	4 300	3 386
Disability pension	6 453 (16)	5 846 (18)	4 380 (15)	3 996 (16)	1 538 (27)	1 368 (30)	535 (12)	483 (14)
Median age	51	52	52	53	50	50	47	48
Living alone	19 337 (49)	15 601 (48)	15 229 (51)	12 279 (51)	2 627 (47)	2 155 (47)	1 477 (34)	1 165 (34)
Low education achievement	9 266 (23)	7 643 (24)	6 254 (21)	5 190 (21)	1 751 (31)	1 438 (31)	1 261 (29)	1 015 (30)
Utilisation of health care	32 249 (81)	32 249 (100)	24 272 (81)	24 272 (100)	4 588 (81)	4 588 (100)	3 386 (78)	3 386 (100)
Public general practitioner		15 233 (47)		10 677 (44)		2 414 (53)		2 139 (63)
Public specialist		14 170 (44)		10 397 (43)		2 104 (46)		1 668 (49)
Private general practitioner		10 453 (32)		8 157 (34)		1 306 (28)		990 (29)
Private specialist		19 197 (60)		14 934 (62)		2 686 (59)		1 577 (47)
								
Men								
Number of countries	140	131	33	32			102	74
Number of individuals	40 471	26 599	30 081	19 708	5 047	3344	5 322	3546
Disability pension	4 618 (11)	3 716 (14)	3 120 (10)	2 542 (13)	956 (19)	743 (22)	542 (10)	431 (12)
Median age	52	52	52	53	50	51	47	48
Alone	19 280 (48)	12 328 (46)	15 510 (52)	9 953 (51)	2 054 (41)	1 327 (40)	1 698 (32)	1 048 (30)
Low education achievement	9 147 (23)	6 344 (24)	6 925 (23)	4 793 (24)	1 201 (24)	833 (25)	1 021 (19)	718 (20)
Utilisation of health care	26 599 (66)	26 599 (100)	19 708 (66)	19 708 (100)	3 344 (66)	3 344 (100)	3 546 (66)	3 546 (100)
Public general practitioner		11 761 (44)		7 978 (40)		1 664 (50)		2 118 (60)
Public specialist		12 231 (46)		8 995 (46)		1 591 (48)		1 645 (46)
Private general practitioner		9 025 (34)		7 136 (36)		963 (29)		926 (26)
Private specialist		9 652 (36)		7 217 (37)		1 299 (39)		1 136 (32)

**Table 2:** Country of birth economic characteristics of disability pension recipients 40 to 64 in Malmo, Sweden, 2003 Country economies according to World Bank classification. Values in parentheses are percentages.

	Disability pension recipients		Country economy					
Women			High income		Middle income		Low income	

	Total	Sought health care	Total	Sought health care	Total	Sought health care	Total	Sought health care

Number of countries	88	86	23	22	16	16	49	48
Number of individuals	6453	5846	4380	3995	1538	1368	535	483
Median age	57	57	58	58	55	55	53	53
Living alone	3 658 (57)	3 300 (56)	2 685 (61)	2 434 (61)	771 (50)	687 (50)	202 (38)	179 (37)
Low education achievement	2 624 (41)	2 319 (40)	1 628 (37)	1 445 (36)	746 (49)	647 (47)	250 (47)	227 (47)
Utilisation of health care	5 846 (91)	5 846 (100)	3 995 (91)	3 995 (100)	1 368 (89)	1 368 (100)	483 (90)	483 (100)
Public general practitioner visit		3 120 (53)		2 108 (53)		713 (52)		299 (62)
Public specialist		3 422 (59)		2 437 (61)		726 (53)		259 (54)
Private general practitioner		1 737 (30)		1 161 (29)		414 (30)		162 (34)
Private specialist		3 350 (57)		2 295 (57)		818 (60)		237 (49)
								
Men								
Number of countries	77	74	20	20	13	13	44	41
Number of individuals	4 618	3 716	3 120	2 542	956	743	542	431
Median age	56	57	57	57	57	57	54	54
Alone	2 877 (63)	2 239 (60)	2 219 (71)	1 741 (68)	456 (48)	344 (46)	20 (37)	154 (36)
Low education achievement	1 841 (41)	1 446 (39)	1 335 (43)	1 052 (41)	329 (34)	261 (35)	177 (33)	133 (31)
Utilisation of health care	3 716 (80)	3 716 (100)	2 542 (81)	2 542 (100)	743 (78)	743 (100)	431 (80)	431 (100)
Public general practitioner		1 790 (48)		1 170 (46)		376 (50)		244 (57)
Public specialist		2 372 (64)		1 714 (67)		400 (54)		258 (60)
Private general practitioner		961 (26)		636 (25)		206 (28)		119 (28)
Private specialist		1 334 (36)		894 (35)		293 (39)		147 (34)

**Table 3:** Multilevel logistic regression analysis, entire population. Probability of receiving disability pensions, individual and contextual effects.

	Women				Men			
Fixed effects	Model A	Model B*	Model C*	Model D*	Model A	Model B*	Model C*	Model D*

		OR (95% CI)	OR (95% CI)	OR (95% CI)		OR (95% CI)	OR (95% CI)	OR (95% CI)

Living alone vs. married		1.72 (1.62–1.82)	1.72 (1.62–1.82)	1.73 (1.63–1.84)		2.64 (2.46–2.83)	2.64 (2.46–2.83)	2.61 (2.43–2.80)
Educational achievement (low vs. high)		2.14 (2.00–2.29)	2.14 (2.00–2.28)	2.19 (2.05–2.33)		2.12 (1.98–2.28)	2.12 (1.98–2.28)	2.09 (1.95–2.24)
Sought health care (yes/no)			2.45 (2.21–2.71)	2.49 (2.27–2.74)			2.09 (1.93–2.27)	2.08 (1.89–2.27
Country economy:								
High income				(reference)				(reference)
Middle income				1.34 (0.82–2.19)				1.61 (1.06–2.44)
Low income				0.88 (0.68–1.14)				1.25 (0.88–1.78)
DIC	34 175	30 118	29 713	30 319	28 150	25 138	24 781	24 684

* Age-adjusted

OR = odds ratio

CI = confidence interval

DIC = deviance information criterion

**Table 4:** Multilevel logistic regression analysis, population who sought health care. Probability of receiving disability pensions, individual and contextual effects for those who sought health care.

	Women				Men			
Fixed effects	Model A	Model B*	Model C*	Model D*	Model A	Model B*	Model C*	Model D*

		OR (95% CI)	OR (95% CI)	OR (95% CI)		OR (95% CI)	OR (95% CI)	OR (95% CI)

Living alone vs. married		1.62 (1.52–1.73)	1.62 (1.52–1.73)	1.63 (1.53–1.74)		2.30 (2.13–2.50)	1.81 (1.50–2.18)	2.30 (2.12–2.50)
Educational achievement (low vs. high)		2.04 (1.91–2.17)	2.04 (1.91–2.17)	2.06 (1.92–2.21)		1.94 (1.79–2.11)	1.94 (1.79–2.11)	1.95 (1.80–2.12)
Public GP visited			1.23 (1.15–1.32)	1.23 (1.15–1.31)			1.09 (1.00–1.18)	1.09 (1.01–1.18)
Public specialist visited			2.11 (1.98–2.25)	2.11 (1.98–2.24)			2.16 (2.00–2.33)	2.16 (2.01–2.32)
Private GP visited			0.95 (0.89–1.02)	0.95 (0.89–1.03)			0.76 (0.69–0.83)	0.76 (0.70–0.83)
Private specialist visited			1.03 (0.96–1.09)	1.03 (0.96–1.07)			1.04 (0.96–1.12)	1.03 (0.96–1.12)
Country economy:								
High income				(reference)				(reference)
Middle income				1.22 (0.75–1.98)				1.58 (1.08–2.33)
Low income				0.76 (0.53–1.07)				1.24 (0.86–1.78)
DIC	29 633	26 390	25 769	26 248	21 151	18 859	18 722	18 722

* Age-adjusted

OR = odds ratio

CI = confidence interval

DIC = deviance information criterion

**Table 5:** Multilevel logistic regression analysis, random effects, entire population.

	Women			Men		
	Country of birth variance (SE)	MOR	DIC	Country of birth variance (SE)	MOR	DIC

Random effects						
Model A (Empty)	0.663 (0.147)	2.17	34 175	0.411 (0.097)	1.84	28 150
Model B*	0.454 (0.101)	1.90	30 118	0.374 (0.092)	1.79	25 138
Random slopes model C*						
Living alone	0.267 (0.072)	1.64	29 664	0.179 (0.072)	1.50	24 684
Married	0.591 (0.113)	2.08		0.388 (0.100)	1.81	
Low educational achievement	0.629 (0.138)	2.13	30 301	0.401 (0.108)	1.83	24 781
High educational achievement	0.390 (0.064)	1.81		0.303 (0.064)	1.69	
Health care visits						
No	0.628 (0.170)	2.13	30 318	0.856 (0.268)	2.42	24 765
Yes	0.449 (0.105)	1.89		0.284 (0.074)	1.66	

* Age-adjusted

SE = standard error

MOR = median odds ratio

DIC = deviance information criterion

**Table 6:** Multilevel logistic regression analysis, random effects, population who sought health care.

	Women			Men		
	Country of birth variance (SE)	MOR	DIC	Country of birth variance (SE)	MOR	DIC

Random effects						
Model A (Empty)	0.561 (0.133)	2.04	29 633	0.273 (0.073)	1.65	21 151
Model B*	0.439 (0.117)	1.88	26 390	0.228 (0.068)	1.60	18 859
Random slopes model C*						
Living alone	0.283 (0.072)	1.66	25 732	0.158 (0.055)	1.46	18 672
Married	0.593 (0.153)	2.08		0.318 (0.094)	1.71	
Low educational achievement	0.572 (0.138)	2.06	25 757	0.254 (0.076)	1.62	18 856
High educational achievement	0.358 (0.096)	1.77		0.241 (0.066)	1.60	
Health care visits						
No public general practitioner visited	0.476 (0.120)	1.93	25 753	0.291 (0.088)	1.63	18 362
Public general practitioner visited	0.428 (0.109)	1.87		0.272 (0.084)	1.63	
No public specialist visited	0.608 (0.148)	2.10	25 718	(no convergence)		
Public specialist visited	0.348 (0.099)	1.76		(no convergence)		
No private general practitioner visited	0.471 (0.123)	1.92	25 760	0.248 (0.080)	1.62	18 353
Private general practitioner visited	0.500 (0.140)	1.96		0.323 (0.111)	1.69	
No private specialist visited	0.387 (0.097)	1.81	25 757	0.254 (0.089)	1.60	18 365
Private specialist visited	0.539 (0.138)	2.01		0.297 (0.076)	1.66	

* Age-adjusted

SE = standard error

MOR = median odds ratio

DIC = deviance information criterion
